# Preparation and properties of chitosan/zein-perilla essential oil composite film: its structure, physicochemical characterization, and antioxidant properties

**DOI:** 10.3389/fnut.2026.1740444

**Published:** 2026-03-16

**Authors:** Zhiyi You, Chao Ai, Shiqi Wen, Yongmei Su, Junping Xu

**Affiliations:** 1 Life Science and Technology School, Lingnan Normal University, Zhanjiang, China; 2 Guangdong Provincial Key Laboratory of Aquatic Product Processing and Safety, Guangdong Province Engineering Laboratory for Marine Biological Products, Guangdong Provincial Engineering Technology Research Center of Seafood, Key Laboratory of Advanced Processing of Aquatic Product of Guangdong Higher Education Institution, College of Food Science and Technology, Guangdong Ocean University, Zhanjiang, China

**Keywords:** antioxidant ability, chitosan, food packing, perilla essential oil, zein

## Abstract

This study used chitosan and zein as film-forming matrices and added perilla essential oil (PEsO) to prepare a new type of fresh-keeping film. The morphology, structure and properties of the composite film were analyzed through scanning electron microscopy (SEM), infrared spectroscopy (FTIR), thermogravimetric analysis (TGA), hydrophilicity/hydrophobicity and antioxidant tests. Compared with chitosan/zein film, films loaded with PEsO exhibited a significant increase in barrier capacity, thermal stability and antioxidant ability. SEM analysis showed that after the addition of PEsO, micropores and particles were formed on the film surface, which affected the mechanical properties of the film and decreased the tensile strength and elongation at break of the film. FTIR indicated that there are inter-molecular interactions between PEsO, chitosan, and zein. Thermo- gravimetric analysis showed that PEsO delayed the thermal decomposition of the film and improved its thermal stability. Overall, the addition of PEsO enabled to improve the optical properties, barrier properties, thermal stability and antioxidant properties of the chitosan/zein film, and the composite film could be a good candidate for food packaging material especially applied for high-fat and easily oxidized foods.

## 1 Introduction

Food packaging extends the shelf life of food by shielding it from external factors such as microorganisms, moisture, and UV rays, thereby playing a crucial role in food processing (1). In recent years, with increasing public concern over food safety and environmental concerns, food packaging has become a significant area of focus. Edible packaging films, made from natural, biodegradable, safe, and non-toxic materials, exhibit favorable mechanical properties and serve as effective barriers to gases. These films inhibit microbial growth, thus reducing food spoilage and oxidation (2). By minimizing adverse reactions (including enzymatic, physical, and chemical reactions) and enhancing thermodynamic and physical barriers that restrict solute transport, water absorption, and oxygen penetration, edible films effectively extend the shelf life of food (3).

Chitosan, a natural polysaccharide and the second most abundant biopolymer after cellulose (4), possesses remarkable film-forming properties, easy availability, and low cost. These attributes make chitosan a promising material for active food packaging and applications. However, its inherent hydrophilicity leads to poor water resistance in pure chitosan-based films, as well as limited antioxidant and antibacterial properties, which restrict its application in antibacterial food packaging (5). Consequently, it is crucial to incorporate complementary biopolymers into chitosan films to improve their overall performance.

Zein is a biodegradable material derived from corn kernels through wet milling, which is generally recognized as safe (GRAS) and low-cost material (2). Due to its rich content of hydrophobic amino acids such as glutamic acid and leucine, while lacking in basic and acidic amino acids, this unique amino acid composition endows corn zein with excellent hydrophobicity and film-forming properties (6). The abundance of hydrophobic amino acid residues further enhances the water vapor barrier performance of zein films. Consequently, zein is an excellent material for forming film. Research indicates that chitosan and zein form a cohesive biopolymer network through intermolecular interactions, which predominantly involve electrostatic attractions between the positively charged amino groups of chitosan and the negatively charged residues of zein, complemented by hydrogen bonding interactions (6). Compared to single-component films, these molecular interactions facilitate the fabrication of composite biopolymer films with superior mechanical integrity and functional properties.

In recent years, incorporating natural active ingredients (such as essential oils and polyphenols) into biodegradable films has emerged as a research hotspot in active packaging (7). Essential oils are secondary metabolites found naturally in various parts of plant, including leaves, bark, stems, roots, flowers or fruits (8). These oils can be extracted from plants and are rich in active ingredients such as phenolic aromatic compounds and terpenes, thereby exhibiting excellent antioxidant and antibacterial properties (9). PEsO is a highly volatile aromatic oily substance obtained from perilla leaves or straw, which has strong antioxidant, antibacterial, and anti-inflammatory activities (10). Compared to other essential oils such as cinnamon, clove and thyme, PEsO offers the advantages of a pleasant aroma and recognized safety profile, making it suitable for widespread use in baked goods, beverages, frozen dairy products, puddings, processed vegetables and soups (11).

Currently, research on preservation films lacks investigation into ternary composite systems comprising chitosan, zein, and perilla essential oil. Such systems offer potential for synergistically combining the film-forming properties of chitosan, the superior water vapor barrier characteristics of zein, and the potent antioxidant activity of PEsO. Accordingly, this study prepared a novel fresh-keeping film using chitosan and zein as the matrix, and embedded with PEsO. The influence of PEsO on the structure and thermal stability of the film was investigated using scanning electron microscope, Fourier infrared spectroscopy, and thermogravimetric analyzer. We hope the present research could provide a theoretical basis for the development and utilization of PEsO, as well as the application of protein-polysaccharide composite films.

## 2 Materials and methods

### 2.1 Materials

Chitosan (degree of deacetylation ≥ 95%) was purchased from Shanghai Macklin Biochemical Technology Co., Ltd. (Shanghai, China); Zein was ordered from Aladdin Reagent (Shanghai) Co., Ltd.; Perilla essential oil was brought from Shanghai Yuanye Biotechnology Co., Ltd.; 1,1-Diphenyl-2-picrylhydrazyl (DPPH) and 2,2′-Azino-bis (3-ethylbenzothiazoline-6-sulfonic acid) (ABTS) were purchased from Shanghai Macklin Biochemical Technology Co., Ltd.

### 2.2 Preparation of films

The preparation of the film was conducted according to the method described by Liming Zhang et al. with minor modifications (12). Specifically, 2.0 g of chitosan was dissolved in 100 mL of a 1% acetic acid aqueous solution and stirred until complete dissolution. Similarly, 2.0 g of zein was dissolved in 100 mL of an 80% ethanol aqueous solution and stirred until fully dissolved. The chitosan and zein solutions were then mixed at a volume ratio of 1:1. Glycerol (0.5 g/100 mL) and Tween 80 (0.4 g/100 mL) were added to the blended solution and stirred for 30 min. Perilla essential oil was added to the uniformly stirred blend solution at concentrations of 0, 1, 2, 3, and 4% (v/v), followed by continued stirring for another 30 min. The mixture was subsequently homogenized at 12,000 rpm for 3 min using a homogenizer. Finally, 15 mL of the film-forming solution was cast onto a plastic plate (10 × 10 × 1.7 cm) and dried at 45 °C for 8 h. All films were left uncovered after being stored at a constant temperature for 48 h. The films containing different concentrations of perilla essential oil (0, 1, 2, 3, 4%) were designated as CZ, CZP1, CZP2, CZP3, and CZP4, respectively.

### 2.3 Scanning electron microscopy

The morphology of the films was observed by scanning electron microscope (SEM, Tescan Mira Lms, Czech) under a 15 kV accelerating voltage and 1,000 × magnification. Films were coated with a thin gold layer before SEM analysis.

### 2.4 Thickness and mechanical properties

The thickness of the film was measured at eight random positions using a micrometer screw gauge (IP64, Sanyo, Japan, 0.001 mm) and the average value was taken. The mechanical properties, including tensile strength (TS) and elongation at break (EB), were determined by a texture analyzer (TA. XT plusC, Shanghai Ruifen Intelligent Technology Co., Ltd., Shang hai, China). The film samples were conditioned at a relative humidity of 50% for 48 h, then sectioned into rectangular strips measuring 1 cm × 7 cm. The distance between clamps and cross-head speed were 50 mm and 5 mm/s, respectively. TS and EB were caculated based on Equation (1) and (2):


$$\mathrm{TS}=\frac{F}{S}$$ (1)

Where, F refers to the maximum tensile force at break (N), and S refers to the cross-sectional area of the film (m^2^).


$$\mathrm{EB}=\frac{L_{1}-L_{0}}{L_{1}}$$ (2)

Where, L_0_ refers to the original length of the film (mm), and L_1_ refers to the length of the film when it breaks (mm).

### 2.5 Fourier transform infrared spectroscopy

The composite film solution was vacuum freeze-dried and ground into powder. A certain amount of potassium bromide powder was added and ground evenly. After successful tablet pressing on the tablet press, the test was carried out promptly. Scanning was conducted in the range of 4,000–400 cm^−1^ by a Fourier transform infrared spectrometer (Bruker Tensor 27, Bruker Corporation Co., Ltd., MA, United States).

### 2.6 Optical properties of films

The transmittance of the film was measured using a UV–vis spectrophotometer (Cary 60, Agilent Technologies Inc., CA, United States). The slit width was 1.0 nm, with a scanning range of 400–800 nm. Baseline correction is performed using air as the reference.

### 2.7 Steady-state fluorescence measurement

The fluorescence spectra of the film-forming solution were obtained using a fluorescence spectrophotometer (RF-5301PC, Shimadzu Corporation, Japan) at an excitation wavelength of 280 nm. The emission wavelength range from 300 to 500 nm was collected at a speed of 100 nm/min (13).

### 2.8 Thermogravimetric analysis

The thermal properties of the film were measured using a synchronous thermal analyzer (STA449F3, Netzsch Co., Ltd., Bavaria, Germany). The sample (about 6.0–7.0 mg) was weighted and put in an aluminum crucible, with an empty crucible as a blank control. The experimental conditions were as follows: the temperature range was 30–600 °C, the heating rate was 10 °C/min, and the nitrogen gas flow rate was 20 milliliters per minute.

### 2.9 Water vapor permeability

Based on the method proposed by Guofeng Zhong et al. with some modifications (14), the water vapor permeability (WVP) of the film was determined through gravimetric analysis. Specifically, the prepared film with a diameter of 1.2 cm was securely affixed to the mouth of a glass bottle containing 10 mL of ultra-pure water. The bottle was subsequently placed in a drying oven maintained at 60 °C. The weight of the glass bottle was recorded at predetermined intervals. The experiment was replicated three times to ensure reliability. WVP was calculated using Equation (3):


$$\mathrm{WVP}(\%)=\frac{W_{0}-W_{t}}{A\times T}\times 100\%$$ (3)

Where, W_0_ and W_t_ refer to the initial and final weights (g) of the glass bottle, respectively; A refers to the cross-sectional area of the bottle opening (m^2^); T refers to the number of days.

### 2.10 Swelling ratio

The swelling ratio of the sample was determined by cutting the film into 2 × 2 cm pieces and weighing it (W_0_). The film was then immersed in 30 mL of distilled water at room temperature for 30 min. After gently wiping off the surface moisture, the film was reweighted (W_t_) (15). Swelling ratio was calculated using Equation (4):


$$\mathrm{SR}(\%)=\frac{W_{t}-W_{0}}{W_{0}}\times 100$$ (4)

### 2.11 Water solubility

The water solubility of the sample was analyzed according to the method described by Chen et al. (16). The film was cut into 2 × 2 cm pieces and dried at 105 °C for 4 h. Subsequently, the initial weight of the film (W_0_) was measured. The film was then immersed in 40 mL of distilled water and gently agitated on a shaker for 24 h. Afterward, the samples were removed from the water, dried again at 105 °C for 4 h, and the final weight (Wt) was measured. Water solubility was calculated using Equation (5):


$$\;\mathrm{WS}(\%)=\frac{\left(W_{0}-W_{t}\right)}{W_{0}}\times 100$$ (5)

### 2.12 Moisture content

The moisture content of the film was determined according to the method proposed by Bhatia et al. (17). After measuring the initial weight (M0), the film (20 × 20 mm) was placed in an oven at 105 °C until a constant weight was achieved. Subsequently, the dried film was weighed (Mt). Moisture content (MC) was calculated using Equation (6):


$$\mathrm{MC}(\%)=\frac{M_{0}-M_{t}}{M_{0}}\times 100$$ (6)

### 2.13 Antioxidant activity

The antioxidant capacity of the films was evaluated by measuring the scavenging ability of DPPH and ABTS free radicals. The film (20 mg) was placed in DPPH solution at room temperature for 30 min. Then, the absorbance value at 517 nm was measured with an enzyme marker (Varioskan LUX, Thermo Fisher Scientific Co., Ltd., America) (18).

For the ABTS assay, the method was performed according to Liu et al. (2) with minor modifications. The film (20 mg) was immersed in 2 mL of ethanol and incubated at room temperature for 24 h. The absorbance of the ABTS solution was then adjusted to 0.7 ± 0.05 with absolute ethanol. Next, 1 mL of the film soaking solution was mixed with 1 mL of the ABTS working solution, and the reaction was carried out in the dark for 6 min. Finally, the absorbance was determined at a wavelength of 734 nm (2). The antioxidation activity was calculated using Equation (7):


$$\text{Antioxidant activity}(\%)=\frac{A_{K}-A_{y}}{A_{k}}\times 100$$ (7)

Where, Ak and Ay refer to the absorbances of the blank group and the sample group, respectively.

### 2.14 Statistical analysis

All the above experiments were conducted in triplicate measurements to ensure accuracy and reproducibility. And the experimental data were analyzed using SPSS 26.0 software (version 26.0, United States) and a significant difference analysis was performed. Figures were drawn using Origin 2019(version: Origin 2021b, United States) and GraphPad Prism (prism 9.5.0, United States).

## 3 Results and discussion

### 3.1 The structure of chitosan/zein-perilla essential oil composite film

**Figure 1:**
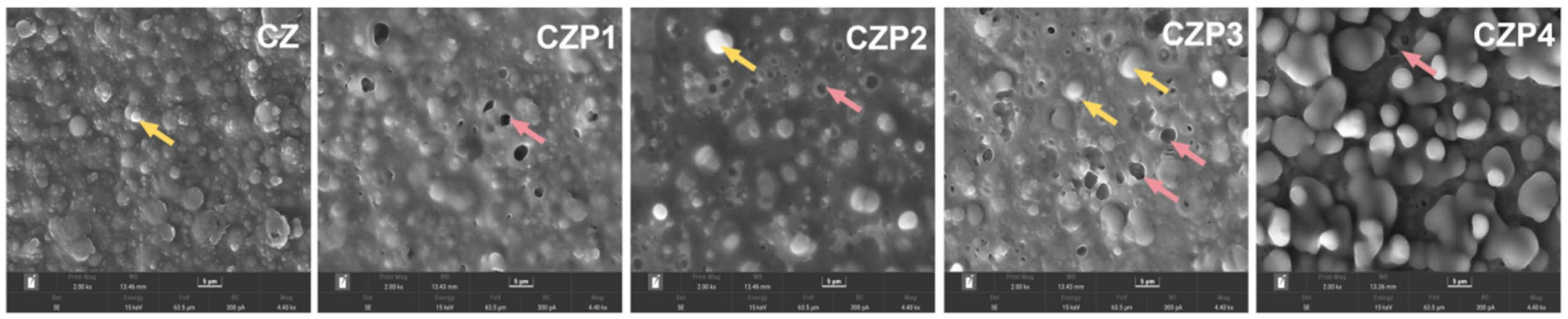
SEM micrographs of composite films loaded with PEsO.

The micro-structure of the film is illustrated in Figure 1. The CZ group film showed good film-forming ability, with a smooth and uniform surface free of cracks. However, after adding PEsO, micropores (indicated by red arrows) and particles (indicated by yellow arrows) appeared on the surface of the film. As the concentration of PEsO increased, both the number and diameter of micropores grew. This phenomenon may be attributed to water evaporation from the film, as well as enhanced emulsification and flocculation of the PEsO during the drying process, resulting in larger oil droplets that form micropores upon further evaporation (19). Similar results have been reported in another study (20), where tiny pores and bubble like structures appeared on the surface following the incorporation of the clove essential oil nanoemulsion. In addition, PEsO, as a hydrophobic compound, is able to rearrange the chitosan-zein network structure, leading to the generation of micro-pore structures (21).

### 3.2 The interaction effect of different PEsO concentrations on chitosan

**Figure 2:**
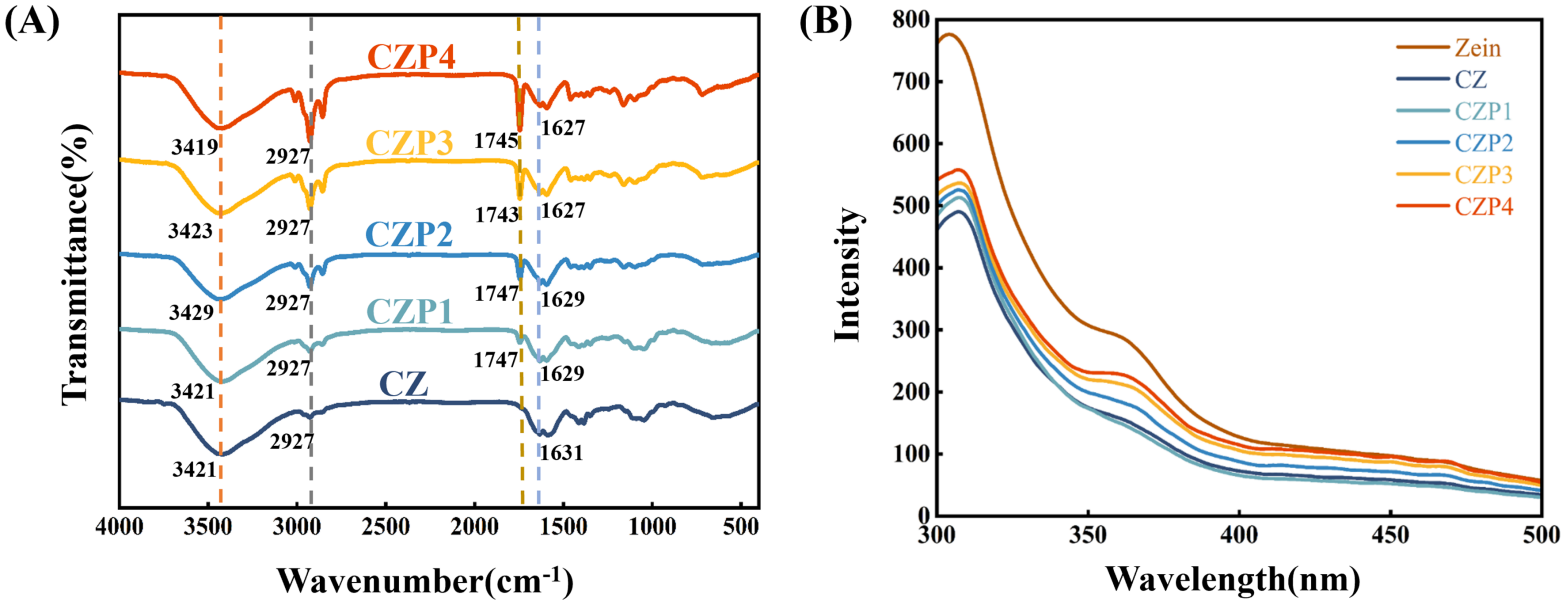
FTIR spectra **(A)** and fluorescence spectrum **(B)** of composite films containing different concentrations of PEsO.

FTIR spectra were used to investigate the interaction effect of PEsO complementary on chitosan. As illustrated in Figure 2A, the infrared spectra of the composite films with varying concentrations of PEsO are presented. A strong and broad spectral band was observed in the region of 3,421 cm^−1^, which was attributed to the stretching vibrations of -OH and -NH groups, indicating the presence of intra-molecular and inter-molecular hydrogen bonding between these functional groups (22).

After the incorporation of PEsO into the film, a new absorption peak appears at 1745 cm^−1^, primarily attributed to the predominant constituent of PEsO-perilla aldehyde-whose aldehyde functional group exhibits characteristic C=O stretching vibrations (23). The intensity of the peak at 2,927 cm^−1^, corresponding to C-H stretching vibration increases with higher essential oil concentration, indicating an enhancement in intermolecular interactions between PEsO and chitosan (24). Similar results have been observed in cassava starch-based edible films containing cinnamon essential oil (25). Furthermore, the peak shift from 1,527 to 1,531 cm^−1^ in amide I region, indicating the possible intermolecular interactions between the -NH of zein and the -OH groups of the essential oil (26).

### 3.3 The interaction effect of different PEsO concentrations on zein

Fluorescence spectroscopy is a powerful tool for investigating molecular interactions between proteins and other components. It is well known that such interactions can induce conformational changes in protein molecules, thereby modulating their intrinsic fluorescence properties. As shown in Figure 2B, the pure zein solution exhibited a fluorescence emission peak at 304 nm, which could be attributed to the presence of tyrosine residues (27). Upon the addition of chitosan, the fluorescence intensity of this peak decreased, suggesting that chitosan may quench the intrinsic fluorescence of zein (13). However, after the incorporation of PEsO, the fluorescence intensity of the emission peak increased as compared to the CZ group. This enhancement may be attributed to the interaction between PEsO, zein, and chitosan, which facilitated the unfolding of zein, leading to conformational relaxation and the exposure of hydrophobic amino acids (28).

### 3.4 Physico-chemical properties of chitosan/zein-perilla essential oil composite film

#### 3.4.1 Thermal properties

**Figure 3:**
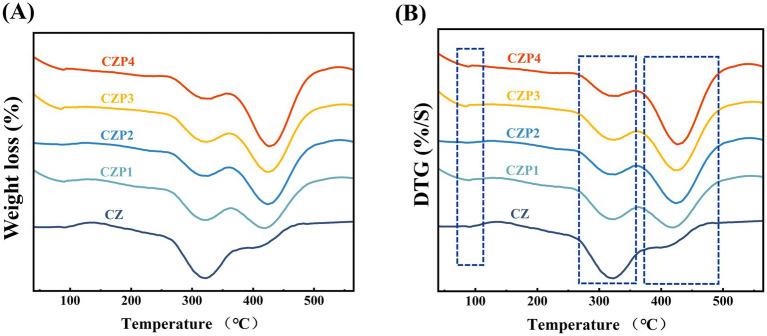
Thermogravimetric analysis **(A)** and the relative derivative curves **(B)** of composite films containing different concentrations of PEsO.

The thermal properties of the composite film reflect its resistance to high temperature decomposition (28). The thermal stability was assessed using thermo-gravimetric analysis (TGA) and derivative thermo-gravimetric (DTG) analysis. As shown in Figure 3A and Table 1, when the weight loss of films reached 5–50%, the temperature corresponding to the same weight loss of the film with PEsO was higher than that of the CZ film. This enhanced thermal stability could be attributed to the presence of essential oils.

**Table 1:** The thickness, tensile strength, and elongation at break of composite films incorporating varying concentrations of PEsO.

Film	Thickness (μm)	TS(MPa)	EB(%)
CZ	38.4 ± 2.1^a^	17.47 ± 0.25^a^	132.15 ± 11.69^a^
CZP1	47.6 ± 2.6^b^	15.72 ± 0.42^b^	122.21 ± 14.40^a^
CZP2	68.6 ± 3.0^c^	8.20 ± 0.38^c^	90.59 ± 8.35^b^
CZP3	85.2 ± 3.0^d^	4.64 ± 0.59^d^	68.64 ± 8.56^c^
CZP4	88.2 ± 2.7^d^	3.58 ± 0.28^e^	68.41 ± 7.11^c^

The results are expressed as the mean ± standard error. Different lower-case letters (a-e) denote significant differences within different samples (*p* < 0.05).

As shown in Figure 3B, the DTG curve indicated that the thermal decomposition of the film proceeded through three main stages. In the first stage, occurring between 70 and 100 °C, the weight loss was primarily due to the evaporation of free water, glacial acetic acid, and other solvents (29). The second stage, from 270 to 360 °C, involved the degradation of chitosan, zein and a portion of PEsO. During this stage, the weight loss rate of the film decreased as the PEsO concentration increased: the CZ film exhibited a weight loss of 34.99%, whereas the CZP1, CZP2, CZP3, and CZP4 films showed weight losses of 26.13, 22.18, 21.41, and 18.13%, respectively. This reduction may be attributed to the strong intermolecular interactions between chitosan zein and PEsO, which delayed the thermal decomposition process (30). The third stage, observed between 360 and 500 °C, was exclusive to films containing PEsO, and is associated with the loss of high-temperature stable components. Notably, this primary weight loss for the CZ film mainly occurred in the second stage, while for films containing essential oils, it predominantly occurred in the third stage. As the concentration of PEsO increased, the weight loss in the second stage decreased, indicating that the addition of PEsO delayed the thermal degradation of the film.

#### 3.4.2 Thickness and mechanical properties

Thickness is an important parameter of the film, which directly affects the performance of the film, such as opacity and mechanical properties. According to Table 2, the film thickness of the CZ group was 38.4 ± 2.07 μm. After adding of 1 mL/g of PEsO, the film thickness increased to 47.6 ± 2.60 μm (*p* < 0.05), and with the increased concentration of the essential oil, the film thickness continued to elevated. According to previous studies, the incorporation of most essential oils tended to increase the thickness of the films, which was primarily due to the rise in the total solid content of the films (31). In addition, after adding the essential oil to the system, some of the essential oil escaped during the drying process, generating fluffy pores, thereby changing the thickness of the film (6), which could be verified from the SEM analysis results.

**Table 2:** Variation in thermal properties of chitosan/zein-perilla essential oil (PEsO) composite films with varying concentrations of PEsO.

Film	T_5%_	T_10%_	T_20%_	T_50%_	Residue(%)
CZ	103.1	202.2	274.1	347.8	22.0
CZP1	210.3	262.5	308.6	402.5	23.5
CZP2	208.3	266.4	315.4	415.1	22.8
CZP3	253.5	295.1	327.3	419.3	23.1
CZP4	273.9	306.9	347.0	427.0	24.7

The tensile strength (TS) is the maximum stress that the film can withstand, while the elongation at break (EB) determines the extent to which the film can be stretched before fracture (32). As shown in Figure 2, the TS of the CZ film was 17.41 ± 0.25 MPa, and the EB was 132.14 ± 11.68%. The incorporation of PEsO into the film results in a significant decrease in TS and EB (*p* < 0.05). This may be because PEsO can easily penetrate into the CS-Zein polymer network, diminishing both intra- and inter-molecular interactions. It thereby weakened the continuity of the film matrix, resulting in the stronger intermolecular polymer interactions being partially replaced by weaker polymer-oil interactions (25). The significant reduction in the EB may be attributed to the incorporation of PEsO filled the pores of chitosan and zein. The strong interactions between perilla oil and the film matrix restricted the mobility of chitosan and zein, thereby decreasing the strain of the film (33). Moreover, the microporous structure formed upon incorporation of essential oils, as observed in SEM, disrupts the continuity of the film surface, thereby affecting its mechanical properties. Similar results have been observed in the research of Zhou et al. (25).

#### 3.4.3 Optical properties

**Figure 4:**
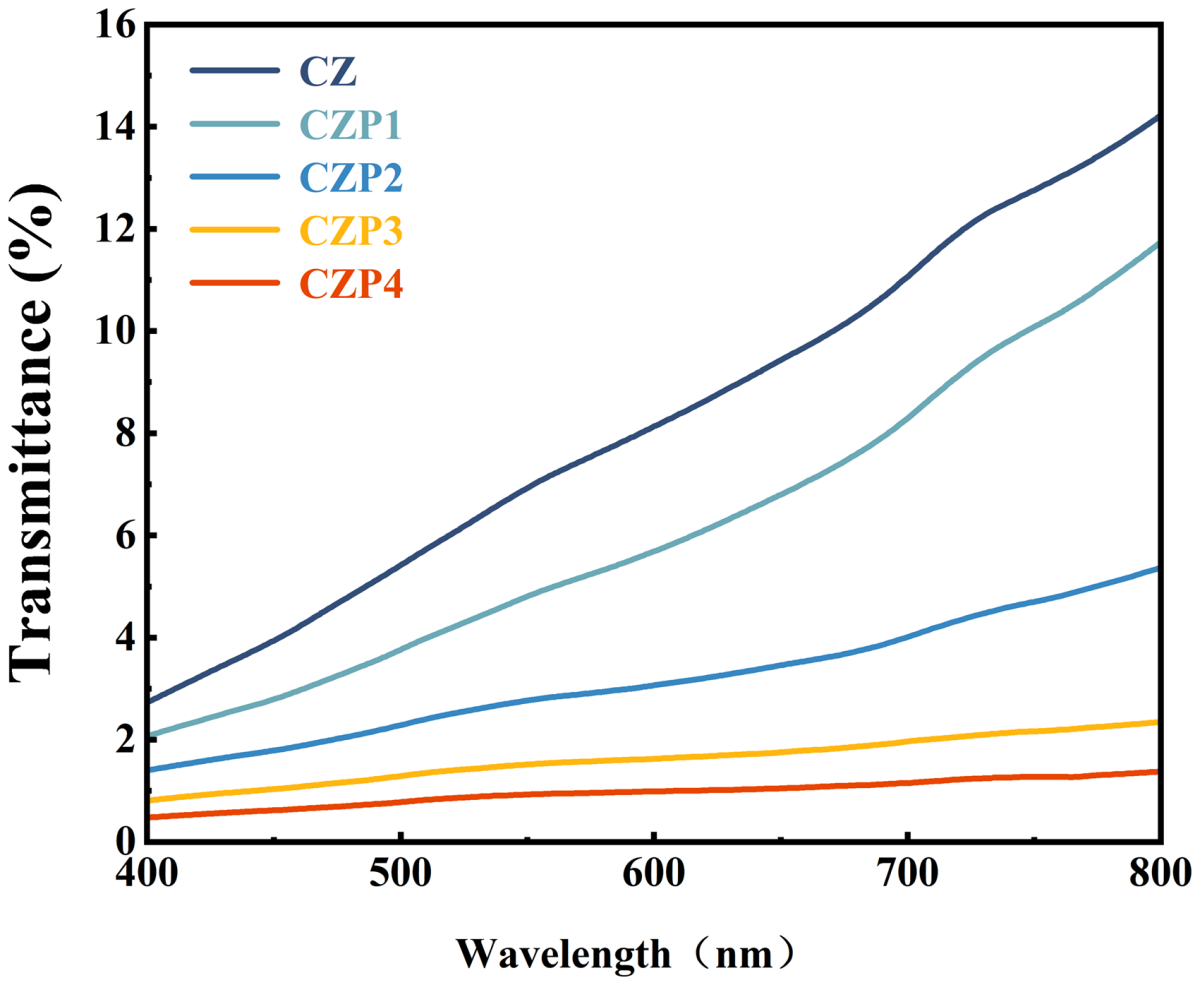
Light transmittance of composite films containing different concentrations of PEsO.

The high opacity of the film indicated its strong UV-blocking properties, which has great advantages for packaging light-sensitive foods (34). As shown in Figure 4, the CZ film exhibited the highest light transmittance, implying the lowest opacity. However, the addition of PEsO reduced the light transmittance of the film decreased. It was found that the decrease in film opacity was positively correlated with the essential oil content, which might be due to the UV radiation-absorbing ability of the phenolic compounds in essential oils (35). Previous studies have reported similar observations when clove essential oil was incorporated into chitosan-based films (20). Moreover, the light scattering, reflection, and coalescence effects caused by PEsO droplets within the film matrix impede channel (18), resulting in a reduction in light transmittance.

#### 3.4.4 Barrier properties

**Figure 5:**
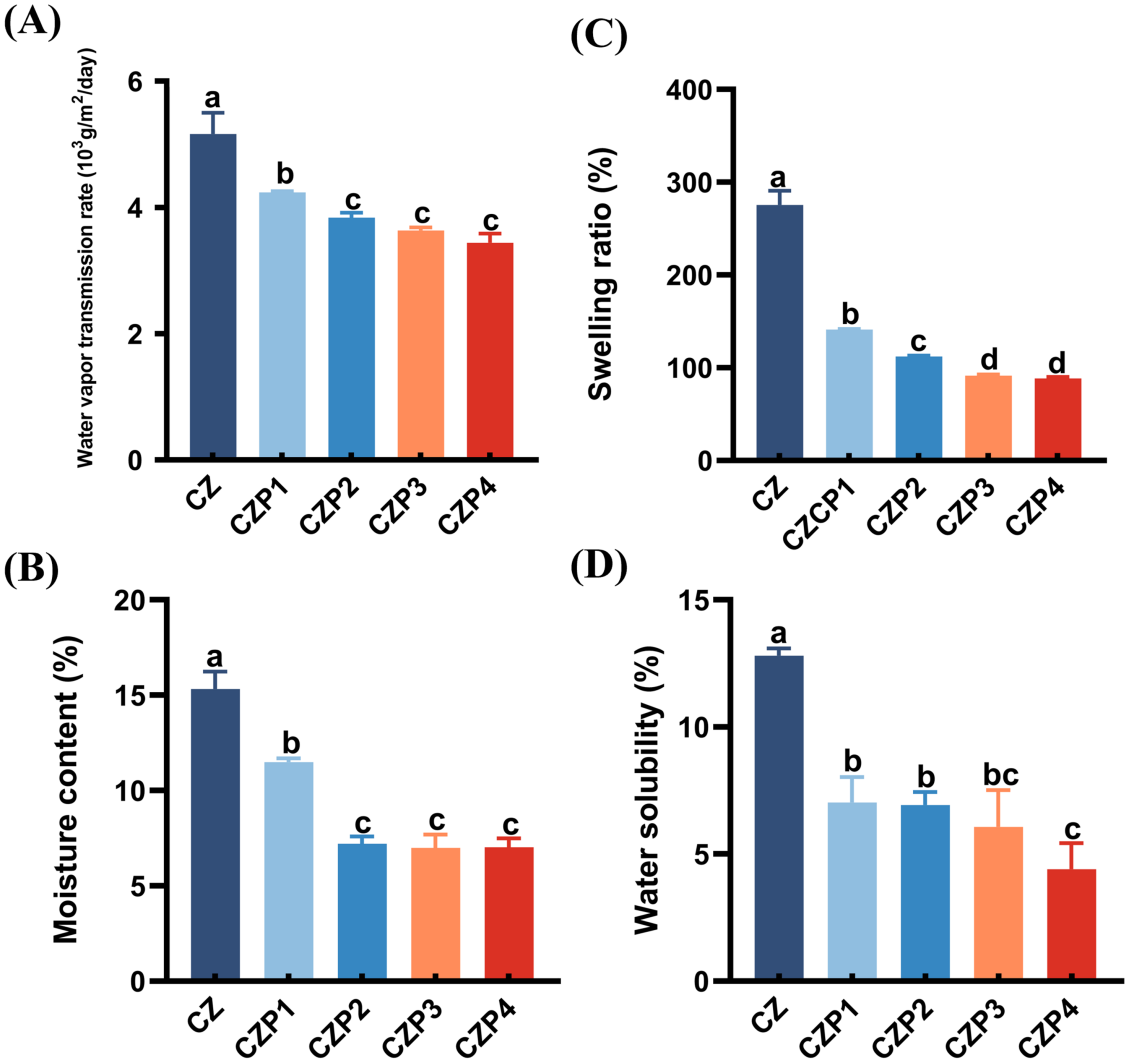
Water vapor transmission rate **(A)**, swelling ratio **(B)**, moisture content **(C)** and water solubility **(D)** of composite films containing different concentrations of PEsO. Data are presented as mean ± SD (*n*=3). Different letters indicate statistically significant differences (*p* < 0.05) as determined by one-way ANOVA followed by Tukey’s multiple comparison test.

Water vapor permeability (WVP) is a critical physical parameter that characterizes the moisture and gas barrier properties of the film separating food from the surrounding atmosphere. A low WVP signifies a superior water vapor barrier performance, thereby effectively blocking moisture exchanges between the food and its surrounding environment (25). As illustrated in Figure 5A, the WVP of the film decreased significantly with increasing PEsO content, indicating a substantial enhancement in waterproof performance following the incorporation of PEsO. This finding aligned with the results reported by Chen et al. (36). The decrease of WVP may be attributed to the hydrophobicity of PEsO and zein, which created a dense and tortuous path direction for water diffusion, resulting in a lower rate of movement of water molecules in the composite film (35).

The swelling ratio is commonly utilized to characterize the water absorption capacity of the film (37). As shown in Figure 5B, the CZ film confirmed the highest swelling ratio of 275.46%, and the addition of PEsO resulted in a significant decrease in the swelling degree of the film (*P* < 0.05). This reduction could be attributed to the strong interactions between PEsO and the film matrix, which diminished the number of hydrophilic groups in chitosan, thereby leading to a decrease in the water absorption capacity of the film (38). This observation was also supported by the FTIR spectra analysis.

The moisture content reflects the total free volume of water molecules within the film network (6). As shown in Figure 5C, the moisture content of the CZ film was 15.32%, while the moisture content of the film significantly decreased (*p* < 0.05) with the addition of PEsO. This reduction could be probably attributed to the hydrophobic nature of PEsO and its interactions with hydroxyl groups in chitosan, which likely decreased the number of hydroxyl groups available for interaction with water molecules, thereby lowering the overall moisture content of the film (16).

For food packaging applications, water solubility (WS) of the film is a critical parameter, particularly in humid environments or when packaging high-moisture foods. Films with lower water solubility tend to exhibit superior performance (39). As shown in Figure 5D, the incorporation of PEsO led to a significant decrease in the water solubility (*p* < 0.05), with WS decreasing by approximately 65% in the CZP4 film compared to the CZ film. This effect was likely due to the hydrophobic interactions generated by the hydrophobic groups in PEsO (38), consistent with observations in sodium caseinate-chitosan films infused with black pepper essential oil (17).

#### 3.4.5 Antioxidant activity

**Figure 6:**
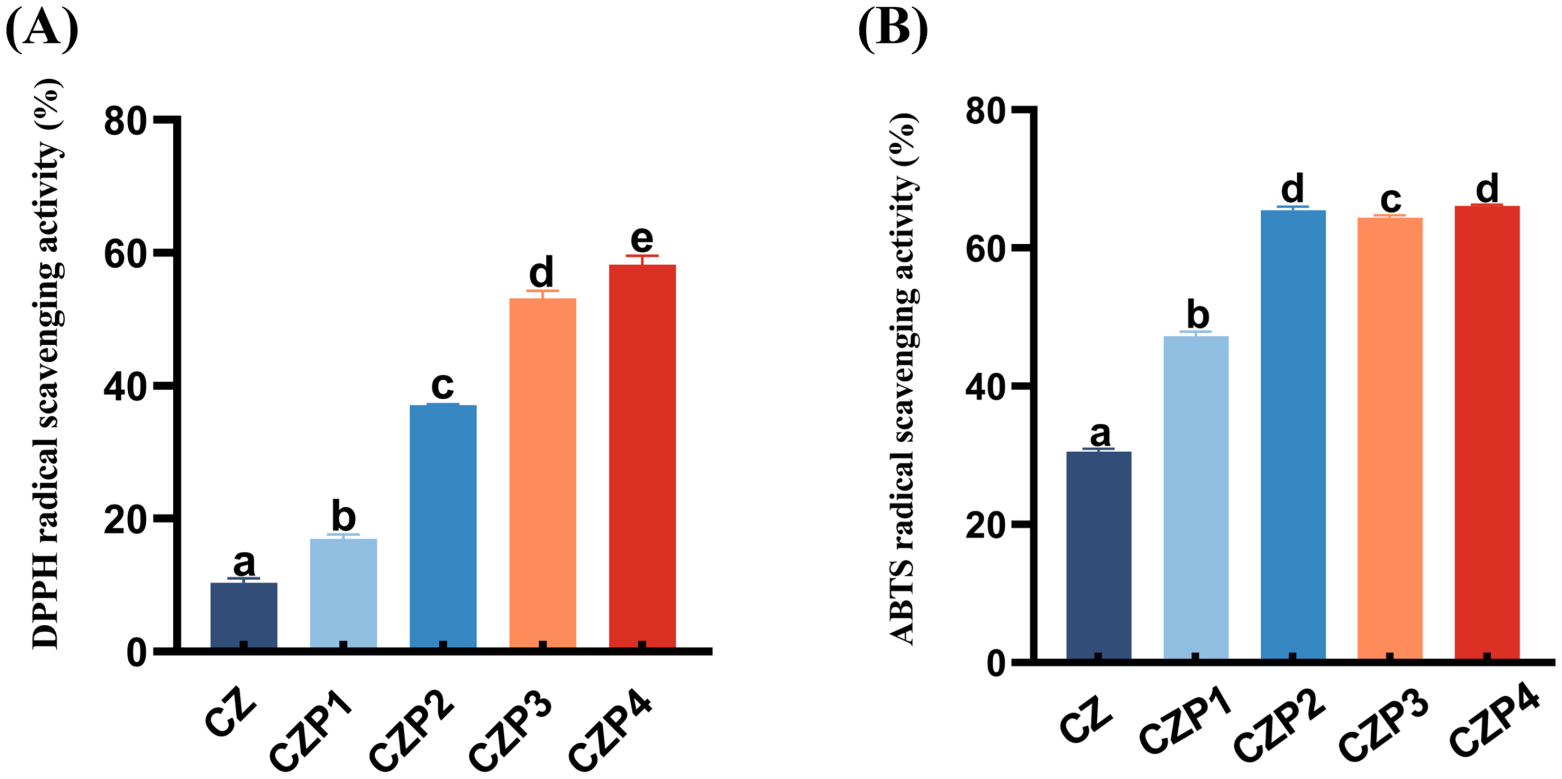
Antioxidant activity of composite films containing different concentrations of PEsO. **(A)** DPPH radical scavenging activity, **(B)** ABTS radical scavenging activity. Data are presented as mean ± SD (*n*=3). Different letters indicate statistically significant differences (*p* < 0.05) as determined by one-way ANOVA followed by Tukey’s multiple comparison test.

The antioxidant activity of food packaging films can significantly influence the duration of food spoilage (40). As shown in Figure 6, the antioxidant activity of the films was evaluated by using DPPH and ABTS radical scavenging assays. Compared to the CZ film, the films containing essential oils exhibited a significant increase in DPPH and ABTS radical scavenging activity. Specifically, the CZP3 and CZP4 films showed markedly higher levels of DPPH and ABTS radical scavenging activity, with statistical significant differences (*p* < 0.05). This enhancement in antioxidant capacity was attributed to the high content of phenolic and aldehyde compounds in PEsO, such as eugenol and perilla aldehyde, which exhibited strong antioxidant properties (41). Furthermore, research conducted by Wang et al. (15) has also demonstrated that the addition of PEsO resulted in an increase in the antioxidant capacity of the film.

## 4 Conclusion

In this study, chitosan/zein composite film loaded with PEsO were successfully fabricated, and the effects of varying concentrations of PEsO on its morphology, thermal stability, barrier properties, optical characteristics, mechanical performance, and antioxidant activity were comprehensively investigated. As the concentration of PEsO increased, the thermal stability and barrier properties of the film improved. However, its tensile strength and elongation at break decreased. Although limited by reduced mechanical performance, this aspect can be addressed in subsequent studies by incorporating crosslinking agents or adjusting the ratio of zein to chitosan, thereby enhancing the mechanical properties of films. Infrared spectroscopy confirmed the presence of PEsO within the composite films, indicating molecular interactions with chitosan and zein. Moreover, the incorporation of PEsO significantly enhanced the antioxidant properties of the films, as evidenced by increases in DPPH radical scavenging activity from 10.37 to 58.90% and ABTS radical scavenging activity from 30.23 to 66.09%. Overall, these findings suggest that chitosan/zein composite films loaded with PEsO possess excellent barrier and antioxidant properties, demonstrating significant potential for extending the shelf life of food products.

## Data Availability

The raw data supporting the conclusions of this article will be made available by the authors without reservation. For any inquiries, please contact the corresponding author, JX, at liluo@naver.com.
